# Design of Tool Shape and Evaluation of Deformation Behavior by Digital Image Correlation Method in V-Bending of Sheet Metal Using Plastic Tools Manufactured by 3D Printer

**DOI:** 10.3390/ma18030608

**Published:** 2025-01-29

**Authors:** Naotaka Nakamura, Yuri Hata, Witthaya Daodon, Daiki Ikeda, Nozomu Adachi, Yoshikazu Todaka, Yohei Abe

**Affiliations:** 1 Department of Engineering, National Institute of Technology (KOSEN), Nagano College, 716 Tokuma, Nagano 381-8550, Japan; 2 Department of Mechanical Engineering, Toyohashi University of Technology, 1-1 Hibarigaoka, Tempaku, Toyohashi 441-8580, Japan; hata@plast.me.tut.ac.jp (Y.H.); ikeda@martens.me.tut.ac.jp (D.I.); n-adachi@me.tut.ac.jp (N.A.); todaka@me.tut.ac.jp (Y.T.); 3 Department of Industrial Engineering, Faculty of Engineering and Technology, Rajamangala University of Technology Isan, Nakhon Ratchasima 30000, Thailand; witthaya.da@rmuti.ac.th

**Keywords:** fused deposition modeling, tool design, deformation behavior, strain distribution, springback

## Abstract

In the V-bending of sheet metals using a pair of plastic punch and die manufactured by a 3D printer, the effects of two different dimensions designed with the same tool geometry on the deformation behaviors of the punch, die, and sheet were evaluated. The deformation behavior and strain distribution of the punch, die, and sheet were analyzed using a digital image correlation method. Sheets from pure aluminum to ultra-high-strength steel were bent using the two tools with different spans; one was designed on the assumption of tool steel material, and the other was designed on the assumption of plastic material. In both tools, the large compressive strain appeared around the center of the punch tip and on the corners of the die. The tools with a long span for the plastic material gave a lower bending force and small deformation of the plastic tools. The angle difference between a bent sheet at the bottom dead center and a tool was smaller for the tools with the long span, although the springback in the bent sheet appeared. It was found that the design method on the assumption of the plastic material is effective for the V-bending plastic tools.

## 1. Introduction

The advancement in additive manufacturing technologies, including 3D printing, that incrementally constructs objects layer by layer based on digital models, has been substantially notable across various industries. This technology has found diverse applications in low-volume production, medical purposes, aerospace, and direct digital manufacturing [1,2]. Additive manufacturing processes offer unique advantages in terms of their material compatibility, resolution, and strength. For instance, binder jetting (BJ), which selectively deposits a liquid binding agent to join powder materials; directed energy deposition (DED), which utilizes focused thermal energy (e.g., laser or electron beam) to fuse materials as they are deposited; stereolithography (SLA), which employs a light source to cure photopolymer resins into three-dimensional objects suitable for high-resolution parts; selective laser melting (SLM), a process that utilizes a laser to melt and fuse metallic and ceramic powders, enabling the creation of fully dense metal parts; and fused deposition modeling (FDM), also known as fused filament fabrication (FFF) which extrudes thermoplastic filaments through a heated nozzle. FDM has gained popularity due to its accessibility and versatility in material selection [3]. These processes enable the manufacturing of mechanical parts without the use of traditional tools. However, the high cost of raw materials and extended lead time required to produce each part make it uncompetitive for large production runs. Despite these limitations, they are suitable for prototyping and producing customized and complex parts, especially in situations where low-volume production and high levels of customization are required [4].

One beneficial application of 3D printing is rapid tooling, which involves the fabrication of molds, dies, and other tooling components used in the production of parts. Unlike the direct manufacturing of mechanical parts, rapid tooling enables the use of 3D printing technologies to create the tools required for traditional manufacturing processes such as injection molding, casting, and sheet metal forming. This approach mitigates the high costs and long lead time typically associated with traditional tool manufacturing. Using 3D printing, the tools can be fabricated from CAD data without skilled labor. Additionally, 3D printing enables the production of complex tool parts in a single piece, eliminating the need for segmentation required in conventional methods [5,6]. Three-dimensional printing of metallic materials using SLM and laser powder bed fusion has been utilized in the fabrication of tools for die casting [7], injection molding [8,9,10], metal forming [11,12,13], and hot extrusion [14]. This technology allows the creation of conformal cooling channels that are difficult or impossible to achieve using conventional methods, leading to improved heat extraction, cooling uniformity, and cycle times, thereby enhancing the overall quality of produced parts.

In hot sheet metal forming, 3D-printed maraging steel for press hardening tools exhibited superior thermal performance compared to conventional AISI H13 tool steel through surface-conforming cooling channels, reducing temperatures, and hot spots [13]. Furthermore, in the stamping processes of the automotive industry, rapid tooling provides tools with excellent performance, facilitating the rapid development of new models, and reshaping of tools. The use of 3D-printed metallic tools produced via the laser powder bed fusion technique, particularly for stamping hot-dip galvanized DP600 steel, significantly reduced lead times and improved material efficiency. However, the initial expenses of these tools remain higher than those of the traditional tools, primarily because of the substantial investment required for laser additive manufacturing equipment and metal powders [11,12]. Although laser-based additive manufacturing is effective for producing tools in metal forming processes, its widespread industrial use is limited by the high costs associated with this technology. To make rapid tooling more accessible and economical, it is crucial to investigate alternative materials and methods.

Plastic-based 3D printing methods, particularly FDM, are increasingly used for rapid tooling applications due to the decreasing prices of FDM machines and the affordability of commonly used filaments like polylactic acid (PLA) and acrylonitrile butadiene styrene (ABS). In the FDM process, a thermoplastic filament is heated and extruded through a nozzle to build a mechanical part layer by layer. The FDM approach has facilitated the rapid development of thermoform tooling [15,16] and has been utilized in injection molding to produce mold inserts, allowing faster tooling production at lower costs than conventional computerized numerical control (CNC) machining methods [17]. Using 3D-printed plastic tools produced by FDM to create flexible and cost-effective production lines for sheet metal forming has demonstrated a reduction in tool production costs compared to traditional methods, such as wire electrical discharge machining and milling, offering environmental benefits owing to recyclability and reducing the need for lubricants [18]. Further studies on V-bending and groove pressing of aluminum sheets found that the plastic tools achieve steady-state conditions with minimal wear after the initial forming strokes. Despite some initial surface changes, the tools were stabilized quickly, and the repeatability of the geometries was good [19]. Studies on FDM for producing sheet metal forming tools indicated that the plastic tools performed well with softer steel DC04 materials, allowing up to 100 parts to be stamped within tolerance, although they were less suitable for harder materials, such as S355MC steel [20].

The feasibility of using 3D-printed plastic tools has been demonstrated across various forming processes. For instance, polyethylene terephthalate (PET) tools could successfully form aluminum sheets without lubrication due to their anti-friction properties, showing minimal wear [21]. Studies on air bending with plastic tools revealed satisfactory precision and repeatability, although there were surface changes after repeated cycles, emphasizing the need for optimized material selection [22]. Selective laser sintering (SLS) was employed to fabricate glass bead-filled polyamide tools for cup drawing, which showed good repeatability and performance for up to 200 cycles [23]. Additionally, the use of plastic tools in deep drawing of sheet metals for small-series car body part production was confirmed to be feasible, although optimization is necessary to meet the dimensional accuracy specifications [24]. In stretch forming, 3D-printed tools made from PLA successfully formed aluminum sheets. Despite the higher forming forces, the tools did not suffer any damage, demonstrating their potential for low-volume production of complex shapes in the aerospace industry, such as aircraft skin [25]. Similarly, FDM-produced tools have proven effective for the volume production of aerospace and architectural panels, further supporting the use of plastic tools in stretch forming applications [26]. Material extrusion (MEX) technologies using PLA and PET demonstrated minimal deformation and good wear resistance in deep drawing applications, ensuring acceptable deviations in the produced parts [27]. The PLA tools were also used in rubber pad forming, maintaining structural integrity under significant forces for low-volume production [28]. Glass fiber-reinforced polycarbonate (GF-PC) tools made via big area additive manufacturing (BAAM) could stamp high-strength steel sheets successfully. Despite a slight reduction in draw depth, the overall lead time and cost savings are significant, making them well-suited for low-volume production [29]. These studies demonstrated that the 3D-printed plastic tools could effectively replace the traditional tools in sheet metal forming processes, particularly for prototyping and low-volume production.

The use of 3D-printed plastic tools presents a promising alternative by substantially reducing manufacturing time and cost [1]. However, plastics inherently possess lower mechanical strength and stiffness compared to metals, raising concerns about their ability to withstand high stresses involved in the metal forming processes, which can result in product inaccuracy and potential damage to the tools [30]. To address these challenges, the mechanical properties of plastic tools are crucial to their performance, with factors such as material selection, layer thickness, and printing parameters playing a significant role in enhancing their mechanical properties and performance. The mechanical properties of four FFF materials used in direct polymer additive tooling, PLA, polycarbonate (PC), polyamide (PA), and polyethylene terephthalate glycol (PETG), were investigated. PLA exhibited the highest flexural and compressive strengths at fine-layer resolution, making it suitable for tool applications. Thinner layers generally increase the mechanical strength [31]. The structural optimization and volume reduction of the plastic tools affect their deformation behavior and wear characteristics. Optimized plastic tools, with up to a 30% reduction in volume, maintained suitable drawing performance and exhibited forming behavior comparable to that of solid plastics and conventional steel tools [32]. Surface analysis after repeated bending cycles showed changes in tool surfaces and dimensional stability, emphasizing the importance of careful material selection and printing strategies [22]. The combination of a steel punch and plastic die, reinforcements, and shape modifications can enhance the performance of these tools in processes such as V-bending and deep drawing [33,34]. To further enhance the mechanical properties and durability of the plastic tools, hybrid systems incorporating metallic bases with plastic tool inserts were developed. These hybrid tools demonstrated comparable performance to traditional metal tools while offering cost reductions and minimizing aesthetic defects [35]. Moreover, the hybrid tools that combine additive manufacturing with ultra-high-performance concrete (UHPC) were investigated for deep drawing applications. These tools, made with a PLA shell filled with UHPC, present a cost-effective solution for low-volume production. Two designs were tested: one with PLA in contact and the other with UHPC in contact with sheet metal. Both configurations showed comparable precision to the steel tools but experienced wear and failure issues. PLA in the contact configuration had high wear owing to the thin shell, whereas UHPC in contact faced early failure. Despite these challenges, hybrid tools offer potential economic benefits [36]. Using 3D-printed plastic composites, such as GF-PC and carbon fiber-filled nylon 12, is feasible for sheet metal forming, enhancing tool strength and wear resistance. The anisotropic properties of these composites significantly affect their performance, making accurate material modeling possible, while finite element simulations can effectively predict the performance of low-cost composite tooling for high-strength steel stamping applications [29,37].

Despite significant progress in utilizing the 3D-printed plastic tools for sheet metal forming, challenges remain in fully understanding their deformation behavior and optimizing their designs for better performance. Considering the mechanical properties and deformation behavior of plastics is essential for developing optimized geometries that minimize the deformation and improve dimensional accuracy. V-bending is one of the most common processes in sheet metal forming, and several studies have applied plastic tools to this process. However, the elastic deformation behavior of all the tools has not been thoroughly observed during actual processing. Measurement techniques, such as digital image correlation (DIC), a non-contact optical method that provides high-resolution, full-field strain measurements [38], are highly effective in capturing detailed deformation data [39]. While DIC has been widely used in material testing and deformation analysis, its application to evaluate the deformation behavior of plastic tools during V-bending remains limited.

In this study, plastic tools manufactured by a 3D printer with the same shape but two different dimensions were analyzed in V-bending with different metallic sheet materials. A punch and die were designed considering the allowable stress of the plastic material of PLA. The designed punch and die were manufactured by the 3D printer, and the deformation behaviors of the punch, die, and sheet during bending were measured by taking photographs. The strain distribution from the photographs was calculated using a DIC method. The deformation behaviors and bending force were discussed.

## 2. Plastic Tool Shape and Bending Conditions

### 2.1. Metallic Sheet Materials and Plastic Material

To evaluate the effect of the bending force in V-bending, a pure aluminum A1100-H sheet, 440 MPa and 590 MPa high-strength steel sheets, and a 980 MPa ultra-high-strength steel sheet were selected as metallic sheet materials. The thickness *t* (mm) of the sheets was 1 mm. The mechanical properties of the sheet metals for V-bending are shown in Table 1. The mechanical properties were measured by a uniaxial tension test based on JIS Z2241 [40]. The *F*-value *F* (MPa) and *n*-value *n* in a power-law expression (*σ* = *Fε^n^* (MPa)) were obtained by a true stress-true strain curve in the tension test. The tensile strength *σ*_B_ (MPa) was from 140 MPa to 1000 MPa. The high *n*-value causes not only a higher bending force but also a wide spreading of the strain in the sheet. Therefore, the deformation behaviors of the punch, die, and sheet are affected by the mechanical properties of the sheets.

**Figure 1:**
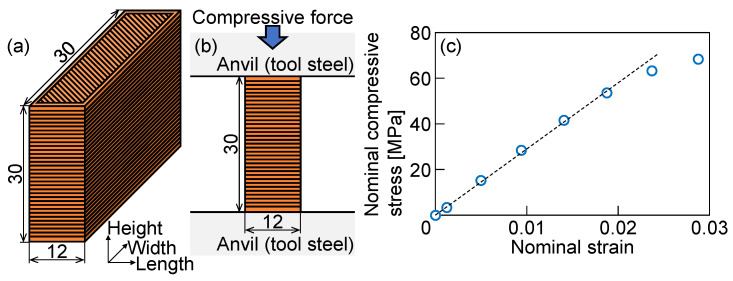
(**a**) Specimen for compression test, (**b**) compression test, and (**c**) nominal stress-strain curve measured from simple compression test of PLA (unit: mm).

A plastic material for tools was PLA, and a 3D printer was FLASHFORGE INVENTOR (Zhejiang Flashforge 3D Technology Co., Ltd., Hangzhou, China), which is a FDM printer. The conditions of 3D printing are shown in Table 2. PLA was printed by each layer with 0.18 mm in thickness on a platform at 50 °C using a nozzle with 0.4 mm in diameter at 200 °C. The numbers of the top, bottom layers and outlines were 3, 3 and 2, respectively. The inside was crosshatched with 80% in infill. Printed plastic material was compressed to identify the mechanical properties of a printed PLA punch and die. The specimen for the compression test, the compression test, and the nominal stress-strain curve measured from the simple compression test of PLA are shown in Figure 1. The dimensions of the specimen were similar to the dimensions in part of the punch. The specimen was compressed until the plastic deformation in 10 mm/min by a pair of parallel tool steel anvils without lubrication using a universal testing machine, Autograph AG-IS (Shimadzu Corp., Kyoto, Japan). The top and bottom surfaces of the specimen were polished with a #600 emery paper to reduce the effect of the deflection of the printed surface shape on the force-stroke curve [31]. The compressive force was measured using a load cell equipped with the universal testing machine. The nominal strain from the instantaneous height of the specimen during the test was calculated using photographs taken by a digital camera. The Young’s modulus *E*_p_ (MPa), yield stress *σ*_y_ (MPa), and Poisson’s ratio of the printed plastic material were 2.9 × 10^3^ MPa, 55 MPa, and 0.33, respectively.

**Figure 2:**
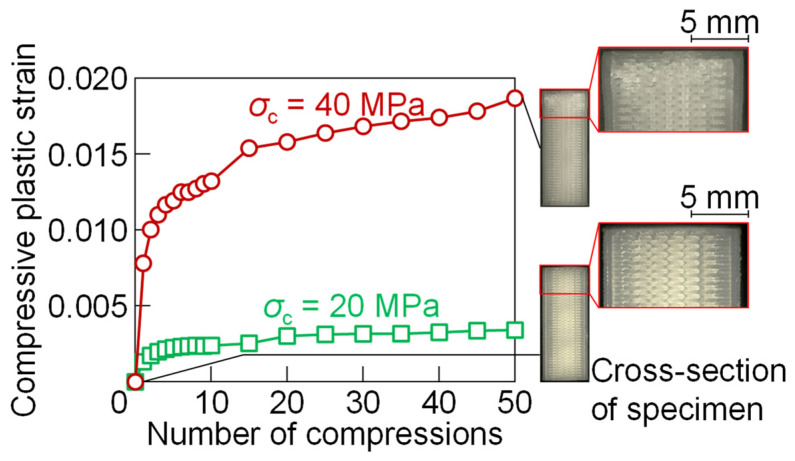
Relationships between compressive plastic strain in height direction and number of compressions in repeated compression test of PLA for *σ*_c_ = 20 MPa and 40 MPa.

In the industry, stamping tools are subjected to repeated forces. The plastic deformation of the printed plastic material under the repeated force was measured by a repeated compression test. The compressive stresses *σ*_c_ = 20 MPa and 40 MPa, i.e., about 36% and 73% of the yield stress obtained from the compression test of PLA, were selected for the 50 times-compression test. The relationships between the compressive plastic strain in the height direction and the number of compressions in the repeated compression test of PLA for *σ*_c_ = 20 MPa and 40 MPa are shown in Figure 2. In both conditions, the increment of the strain until 10 times was large, and then the increment was reduced. The plastic strain in *σ*_c_ = 40 MPa was larger than that in *σ*_c_ = 20 MPa, and the local plastic deformation at the top of the cross-section of the specimen 50 times was observed due to the collapse of the voids between the plastic layers.

### 2.2. Tool Shape Design Method and Bending Conditions

Bending tool shapes using PLA, which has a lower yield stress than the sheet metals, were designed. In order to bend the sheet metals, a series of assumptions have been given.

(1) The bending angle is 90°.
(2) The radius of the bending corner is generally three times the sheet thickness *t* (mm) over the minimum bending radius at least. Thus, the punch corner radius is 3 mm.
(3) To meet the plane strain state conditions in bending, 30 mm in width *b* (mm) of the sheet is selected, which is 30 times the sheet thickness *t* (mm).
(4) The punch and die widths are the same as the sheet width *b* (mm), and the punch length is the same as the span *W* (mm) in bending.

The span *W* (mm) in bending was designed under the allowable punch pressure *p*_a_ (MPa). To select the span *W* (mm), at first, the maximum free bending force *P*_e_ (N) was estimated, and then the allowable punch pressure *p*_a_ (MPa) was used. The bending force is a force causing bending, i.e., the force applied to a sheet during the V-bending process to achieve plastic deformation and form the desired bend angle. This force includes contributions from elastic and plastic deformations of the sheet, and the maximum free bending force *P*_e_ (N) [41] in V-bending is given in

(1) $$P_{e}=1.33\sigma_{B}\frac{bt^{2}}{W}.$$

The sheet width *b* = 30 mm was used as the assumption. The tensile strength of the sheet $\sigma_{B}$ (MPa) and the sheet thickness *t* (mm) in Table 1 were used. The mean punch pressure *σ*_p_ (MPa) is given in

(2) $$\sigma_{p}=\frac{P_{e}}{Wb}.$$

From Equations (1) and (2), the span *W* (mm) is given in

(3) $$W=\sqrt{\frac{{1.33\sigma }_{B}t^{2}}{\sigma_{p}}}.$$

**Figure 3:**
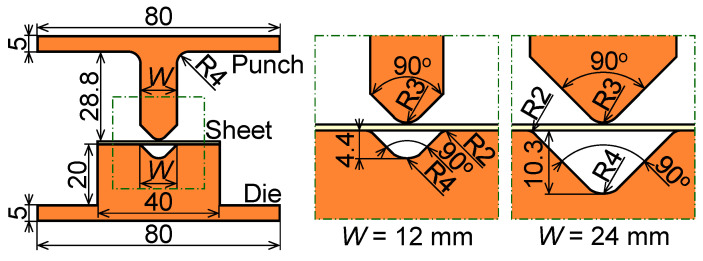
Dimensions of plastic tools and sheet for V-bending (unit: mm).

Assuming that two allowable punch pressures *p*_a_ = *σ*_p_ = 10 MPa and 2.5 MPa for bending the 980 MPa steel sheet, i.e., about 20% and 5% of the yield stress of PLA, the spans *W* = 12 mm and 24 mm were selected. The estimated maximum free bending force and mean punch pressure in V-bending are shown in Table 3. The ratio of the yield stress of PLA to the mean punch pressure was from approximately 6 to 180. The dimensions of the plastic tools and sheet for V-bending are shown in Figure 3. The bending tests were performed using the universal testing machine Autograph AG-IS (Shimadzu Corp., Kyoto, Japan). The bending force was measured using the load cell equipped with the universal testing machine.

### 2.3. Evaluation of Deformation Behavior Using Digital Image Correlation Method

**Figure 4:**
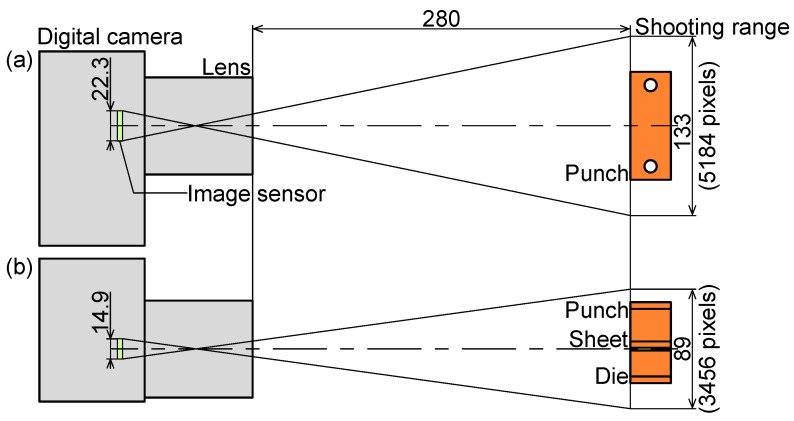
Image sensing conditions for observing deformation of tools and bend angles of sheet in V-bending using digital camera. (**a**) Top view and (**b**) front view (unit: mm).

The image sensing conditions for observing the deformation of the tools and bend angles of the sheet in V-bending using the digital camera are shown in Figure 4. To measure the deformations of the punch and die during bending, photographs of the sidewall surfaces of the tools and sheet were taken with a digital camera EOS 60D (Canon Inc., Tokyo, Japan) with a lens SP AF 60 mm F/2 Di II LD [IF] MACRO 1:1 (Tamron Co., Ltd., Saitama, Japan). The working distance was approximately 280 mm. The image sensor is 22.3 mm × 14.9 mm CMOS. The effective pixels of the image sensor are approximately 18 megapixels; therefore, the resolution was about 26 μm/pixel. The taken images were used to understand the strain distribution by a DIC software 2D-DIC DIPP-Strain 2D Ver. 1.3.5.0 (Kato Koken Co., Ltd., Isehara, Japan). To increase the contrast in the image, the sidewall surfaces of the punch and die were painted in a random pattern with black and white. The size of the subset and the gauge length to calculate the strain were 21 pixels × 21 pixels and 30 pixels, respectively.

## 3. Bending Results

### 3.1. Deformation Behaviors of Tools and Sheet in V-Bending

**Figure 5:**
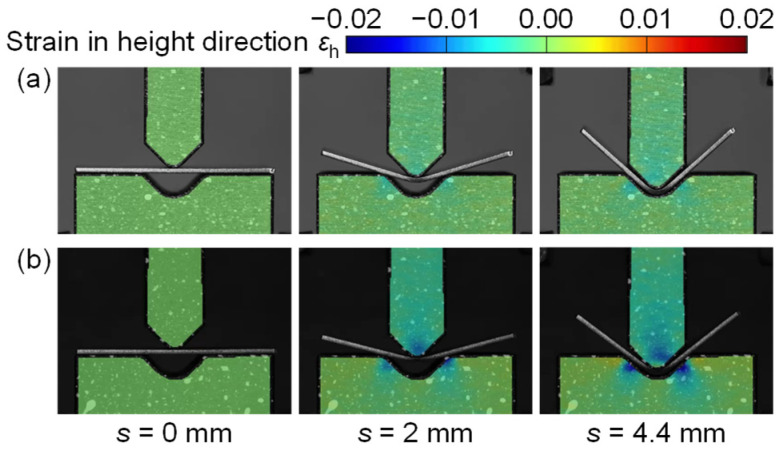
Strain distributions of punch and die in height direction in bending of (**a**) 440 MPa and (**b**) 980 MPa steel sheets for *W* = 12 mm.

The strain in the height direction *ε*_h_ was calculated using the DIC software from the photographs of the sidewall surfaces of the punch and die. The strain distributions of punch and die in the height direction in bending of the 440 MPa and 980 MPa steel sheets for *W* = 12 mm are shown in Figure 5, where *s* (mm) is the punch stroke in bending. Although the DIC method is generally used for measuring large deformations such as the plastic strain, the elastic strain of the punch and die could be measured due to the low Young’s modulus of PLA. For the 440 MPa steel sheet during bending, a large compressive strain was observed in the punch tip and die corners. For the 980 MPa steel sheet, the larger compressive strain was observed, and the maximum compressive strain was about −0.02, and then the strain reached the strain in the yield stress in Figure 1. After the test, the plastic deformation in the punch tip and die corners appeared for the 980 MPa steel sheet.

**Figure 6:**
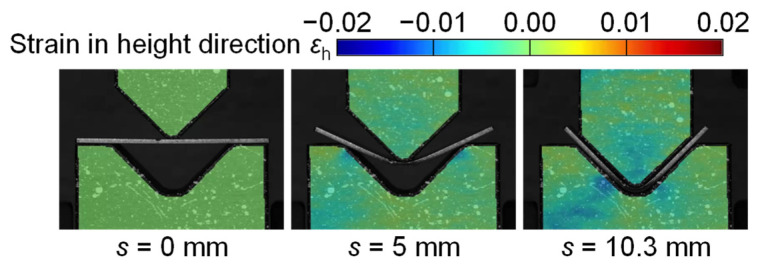
Strain distributions of punch and die in height direction in bending of 980 MPa steel sheet for *W* = 24 mm.

The strain distributions of punch and die in the height direction in bending of the 980 MPa steel sheet for *W* = 24 mm are shown in Figure 6. Although the distribution of strain is similar to the distribution for *W* = 12 mm in Figure 5, the strain concentration eases. The maximum compressive strain was about −0.01. Thus, it is about half of the strain for *W* = 12 mm and is in the elastic deformation. The deformation of the tools was reduced by the increased span. As shown in Figure 5 and Figure 6, the strain distributions in the punch and die depending on the force were observed by the DIC method.

**Figure 7:**
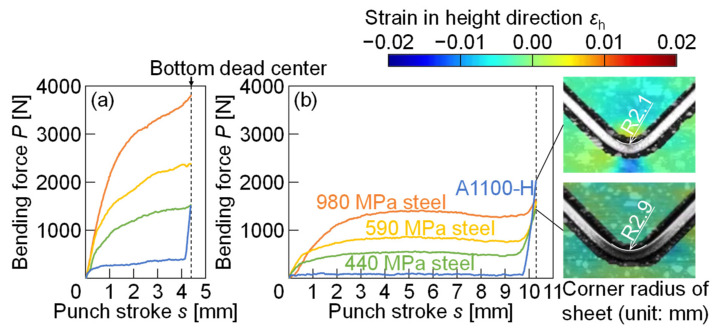
Bending force-punch stroke curves for *W* = (**a**) 12 mm and (**b**) 24 mm.

The bending force-punch stroke curves for *W* = 12 mm and 24 mm are shown in Figure 7. The forces for *W* = 24 mm were lower than those for *W* = 12 mm, whereas the stroke of the slide for *W* = 24 mm was longer than that for *W* = 12 mm. The force increased with the increase in punch stroke and tensile strength of the sheet. The maximum forces appeared at the bottom dead center. The forces for the A1100-H sheet in *W* = 12 mm and 24 mm rapidly increased around the bottom dead center. This is caused by bottoming with the smaller radius of the sheet bend corner than the punch corner radius [42]. The small radii of the A1100-H sheets for *W* = 12 mm and 24 mm were caused by the concentration of the deformation around the sheet bend corner during bending due to the small work-hardening exponent of *n* = 0.042. As shown in this result, not only the strain distribution in general bending but also the strain distribution in bottoming was observed by the DIC method.

### 3.2. Bend Angle of Sheets

**Figure 8:**
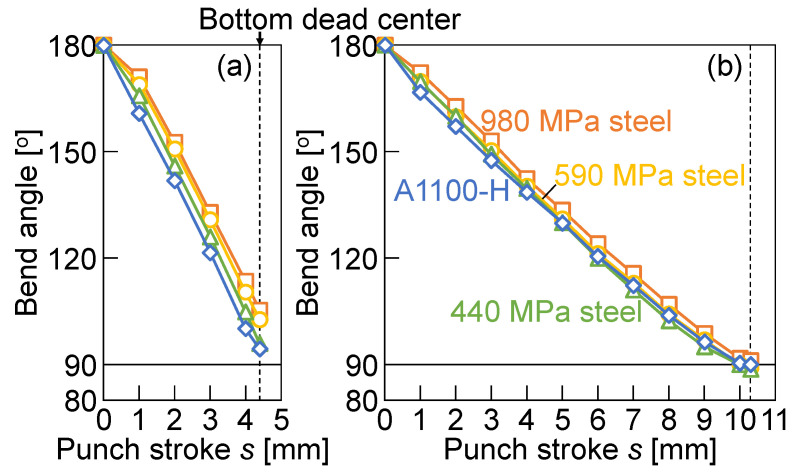
Variations in bend angle with punch stroke for *W* = (**a**) 12 mm and (**b**) 24 mm.

The variations in the bend angle with the punch stroke for *W* = 12 mm and 24 mm are shown in Figure 8. The bend angles of all the sheets decreased to 90° with increasing the punch stroke. Although the bend angles at the bottom dead center for *W* = 12 mm increased with increasing the tensile strength of the sheet, those for *W* = 24 mm were around 90°.

**Figure 9:**
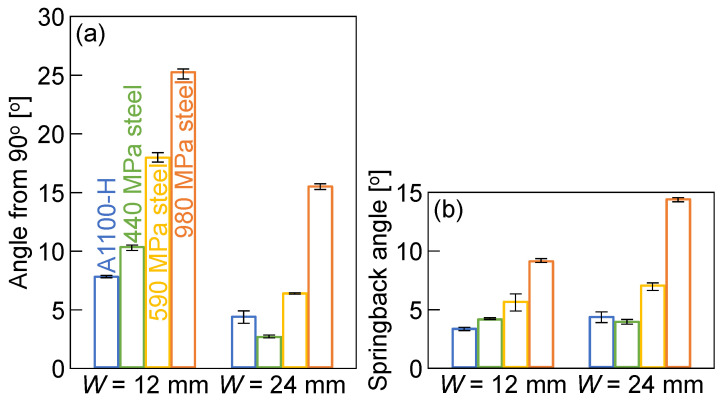
(**a**) Angle from 90° and (**b**) springback angle of bent sheets for *W* = 12 mm and 24 mm.

A bent sheet was taken out from the tools after bending, and then the bend angle of the sheet was measured. The angle from 90° and the springback angle of bent sheets for *W* = 12 mm and 24 mm are shown in Figure 9. The springback angle was obtained by the difference between the measured bend angle of the sheet and the bend angle at the bottom dead center in Figure 8. Both the bend angle and the springback angle increased with increasing the tensile strength of the sheet. The bend angles for *W* = 24 mm were smaller than those for *W* = 12 mm because of the reduced deformation of the punch and die. Both of the springback angles for *W* = 12 mm and 24 mm were almost similar. Because the bend angles in Figure 9a and springback angles in Figure 9b for *W* = 24 mm are almost similar, it seems that the bend angles at the bottom dead center for *W* = 24 mm were almost 90°. The results are reasonable, as shown in Figure 8b.

### 3.3. Strain Distributions of Tools

**Figure 10:**
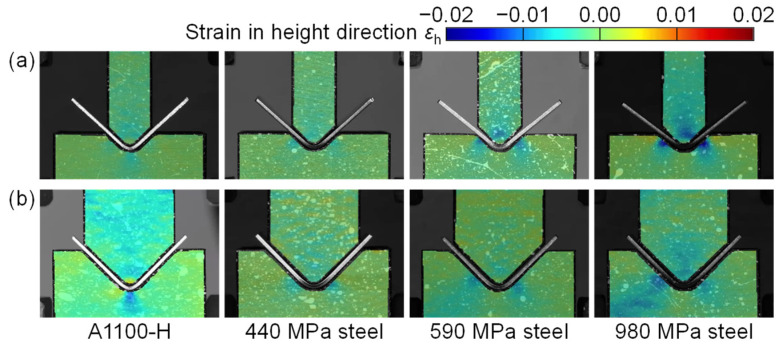
Strain distributions of punch and die in height direction at bottom dead center for *W* = (**a**) 12 mm and (**b**) 24 mm.

The strain distributions of punch and die in the height direction at the bottom dead center for *W* = 12 mm and 24 mm are shown in Figure 10. The strain in the punch was almost uniform in the parallel part, i.e., the distribution appeared around the tip. The large compressive strain appeared around the center of the punch tip. In the die, the large compressive strain appeared on the inclined angles for the steel sheets, whereas the large strain for the A1100-H sheet appeared on the bottom center by contacting the sheet bend corner with the small radius. Therefore, bending with bottoming was caused in the A1100-H sheet. This result is reasonable due to the same tendency in the bending force, as shown in Figure 7. The maximum compressive strain appeared in the 980 MPa steel sheet for *W* = 12 mm, and the value was about −0.02. The value reached the plastic deformation in Figure 1. In the other conditions, the plastic deformation of the tool was not observed.

**Figure 11:**
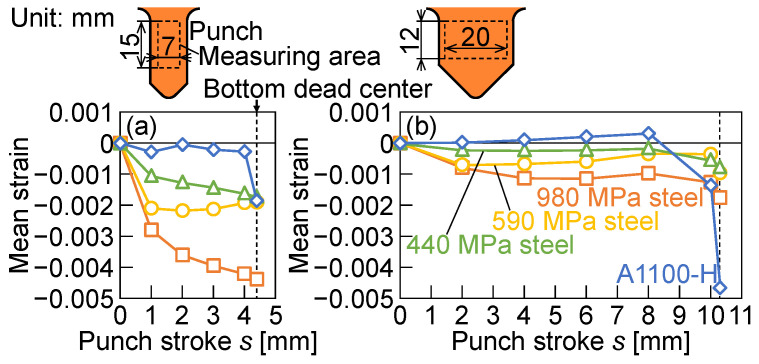
Relationships between mean strain of punch in height direction and punch stroke for *W* = (**a**) 12 mm and (**b**) 24 mm.

The relationships between the mean strain of the punch in the height direction and the punch stroke for *W* = 12 mm and 24 mm are shown in Figure 11. The measuring area is shown in the figure. The maximum compressive strain appeared at the bottom dead center. The sharp increment of the compressive strain in the A1100-H sheet appeared due to bottoming, as shown in Figure 7 and Figure 10 for both spans. The maximum compressive strain in the steel sheets for *W* = 24 mm was lower than that for *W* = 12 mm. The maximum compressive strains were about −0.005 for *W* = 12 mm in the 980 MPa steel sheet and *W* = 24 mm in the A1100-H sheet, respectively; thus, it seems that the values are in the elastic deformation from Figure 1. It is noted that the large strain in the punch was caused not only by the high strength of the sheet but also by the deformation behavior of the sheet, as shown in the A1100-H sheet.

## 4. Discussion

### 4.1. Bending Force

**Figure 12:**
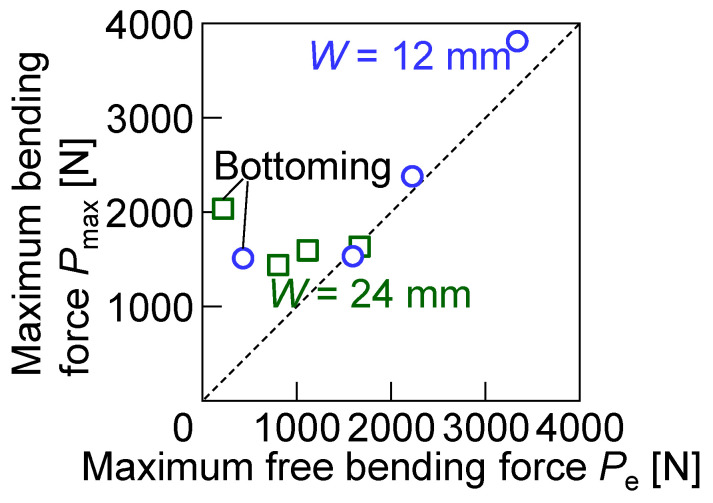
Relationship between maximum bending force and maximum free bending force.

The maximum bending force *P*_max_ (N) from the experiment and the maximum free bending force *P*_e_ (N) from Equation (1) are compared in Figure 12. The maximum bending force was almost the same as the force from Equation (1), except for bending with bottoming. The discrepancy between the measured and empirical forces under bottoming conditions arises primarily from factors not accounted for in Equation (1). During bottoming, the corner radius of the sheet becomes smaller than the punch corner radius, significantly increasing the contact stress and introducing deformation in the contact region, such as a reference [43]. Therefore, in the bending conditions with bottoming, the measured force was larger than the force from Equation (1). It is noted that Equation (1) gives an underestimation of the force in bending with bottoming.

### 4.2. Bend Angle and Springback Angle of Sheets

**Figure 13:**
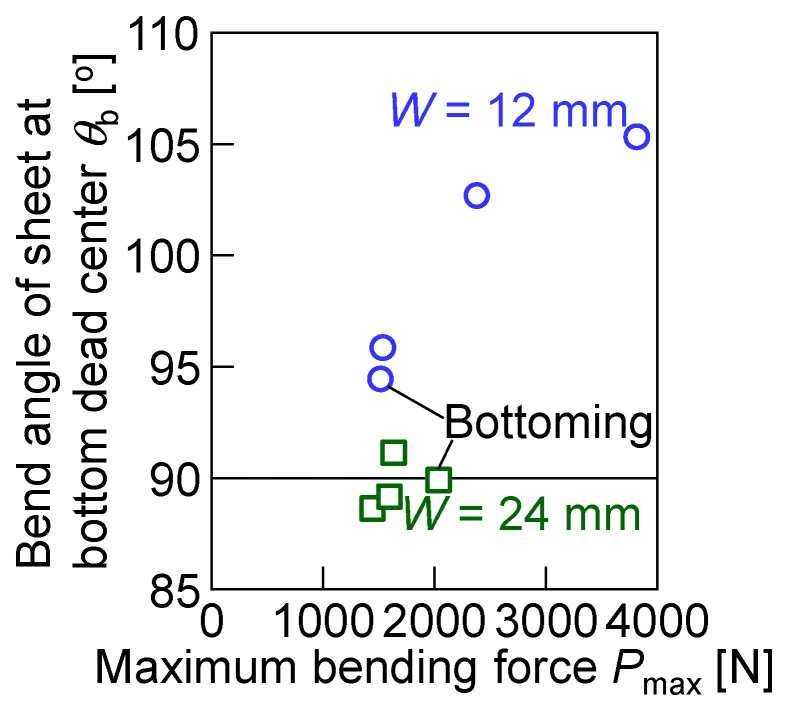
Relationship between bend angle of sheet at bottom dead center and maximum bending force.

The relationship between the bend angle of the sheet at the bottom dead center and the maximum bending force is shown in Figure 13. The bend angle of the sheet at the bottom dead center for *W* = 12 mm was larger than that for *W* = 24 mm. The bend angle increased with increasing the maximum bending force for *W* = 12 mm. This tendency was caused by the increased deformation of the punch tip with the small contact area and large bending force, as shown in Figure 7 and Figure 10. The bend angle for *W* = 24 mm with the larger contact area and smaller bending force was about 90°.

**Figure 14:**
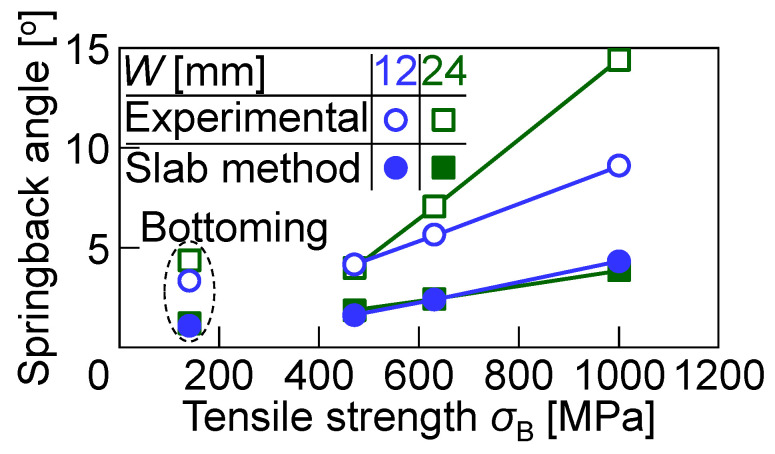
Relationships between springback angle of bent sheet and tensile strength of sheet for *W* = 12 mm and 24 mm.

The relationships between the springback angle of the bent sheet and the tensile strength of the sheet for *W* = 12 mm and 24 mm are shown in Figure 14. The calculated springback angle Δ*θ*_c_ (°) by a slab method [44] is given in

(4) $$∆\theta_{c}=\frac{3F}{\left(n+2\right)E}{\left(\frac{2\rho }{t}\right)}^{1-n}\theta_{b},$$

where *E* (MPa), *ρ* (mm) and *θ*_b_ (°) are the Young’s modulus, the measured corner radius and the bend angle of the sheet at the bottom dead center, respectively. The *F*-value *F* (MPa), the *n*-value *n*, and the sheet thickness *t* (mm) in Table 1 were used. Each of the Young’s modulus for the steel and aluminum sheets is 206 × 10^3^ MPa and 70 × 10^3^ MPa, respectively, although the Young’s modulus of the sheet after the plastic deformation decreases [45]. The springback angle increased with increasing the tensile strength of the sheet, except for bending with bottoming. Although the tendency of the slab method was similar to the experimental results, the angles were lower than the experimental ones. Not only does the reduced Young’s modulus of the sheet after the plastic deformation influences the springback angles, but the stress distributions in the sheet under the elastically and plastically deformed punch and die also contribute significantly to increasing the springback angles. These complex interactions deviate from the assumptions of the slab method, which considers idealized boundary conditions and neglects the elastic compliance of the tools. The prediction of springback angles, particularly for sheets formed with bottoming or with tools manufactured from low-stiffness materials such as PLA, remains a challenging area. The elastic deformation of the punch and die alters the contact stress distribution and effective bending radius, leading to discrepancies between the observed and predicted values. In addition, localized stress concentrations and plastic strain during bottoming introduce further deviations. For more accurate predictions of the springback angles, particularly in bending with plastic tools, a finite element simulation incorporating the elastic-plastic properties of the punch, die, and sheet is essential.

## 5. Conclusions

In this study, the plastic tools manufactured by the 3D printer with the same shape but two different dimensions were designed and evaluated in V-bending with different metallic sheet materials. The strain distributions on the sidewall surfaces of the punch and die in bending were calculated using the DIC method. Based on the results obtained, the following conclusions were drawn:

(1) The large compressive strain occurred around the center of the punch tip and the inclined angles of the die during general bending, whereas bending with bottoming resulted in the compressive strain concentrated on the bottom center. The ability of the DIC method to capture the strain distribution in both general bending and bottoming conditions was effectively demonstrated.
(2) The experimental maximum bending force closely matched the theoretical free bending force under non-bottoming conditions, thereby validating the accuracy of the empirical model in such cases. However, in bottoming, the experimental forces exceeded the predicted values due to additional constraints and localized stress concentrations.
(3) Increasing the span of the tools reduced the bending force under conditions without bottoming and minimized the elastic deformation of the punch and die. This reduction in tool deformation allowed the bent sheet angle at the bottom dead center to align closely with the designed tool angle.
(4) The springback angle increased with the tensile strength of the sheet. Modifying the tool angle based on the estimated springback angle becomes a feasible method for achieving the desired product angles, particularly in 3D-printed plastic tools.

However, the mechanisms underlying springback when plastic punches and dies are employed remain complex and require further investigation. Future work should focus on developing finite element simulations and experimental methodologies to better understand and predict the springback behavior, considering the elastic and plastic deformation of the tools and their interaction with various sheet materials. In addition, investigating the long-term durability and wear resistance of 3D-printed plastic tools under repeated forming conditions would further enhance their applicability in industrial settings.

## Figures and Tables

**Table 1:** Mechanical properties of sheet metals for V-bending.

Sheet	Thickness *t* [mm]	Tensile Strength $\sigma_{B}$ [MPa]	Proof Stress [MPa]	Elongation [%]	*F*-Value *F* [MPa]	*n*-Value *n*
A1100-H	0.96	140	130	4.8	160	0.042
440 MPa steel	1.01	470	300	30	800	0.20
590 MPa steel	1.03	630	470	24	1060	0.19
980 MPa steel	1.00	1000	680	15	1430	0.11

**Table 2:** Conditions of 3D printing.

Nozzle Diameter [mm]	Temperature [°C]		Layer Thickness [mm]	Number		Infill [%]
	Nozzle	Platform		Top and Bottom Layers	Outlines	
0.4	200	50	0.18	3	2	80

**Table 3:** Maximum free bending force and mean punch pressure in V-bending.

Sheet	Span *W* = 12 mm			Span *W* = 24 mm		
	Maximum Free Bending Force *P*_e_ [N]	Mean Punch Pressure *σ*_p_ [MPa]	*σ*_y_/*σ*_p_	Maximum Free Bending Force *P*_e_ [N]	Mean Punch Pressure *σ*_p_ [MPa]	*σ*_y_/*σ*_p_
A1100-H	429	1.19	46.2	215	0.299	184
440 MPa steel	1590	4.42	12.4	797	1.11	49.5
590 MPa steel	2220	6.17	8.91	1110	1.54	35.7
980 MPa steel	3330	9.25	5.95	1660	2.31	23.8

## Data Availability

The original contributions presented in the study are included in the article. Further inquiries can be directed to the corresponding authors.
